# Deep learning-based methods for phenotypic trait extraction in rice panicles

**DOI:** 10.3389/fpls.2025.1730366

**Published:** 2026-02-12

**Authors:** Zhiao Wang, Ruihang Li, Wei Li, Xiaoding Ma, Shen Yan, Maomao Li, Binhua Hu, Ming Tang, Guomin Zhou, Jian Wang, Jianhua Zhang

**Affiliations:** 1 Agricultural Information Institute, Chinese Academy of Agricultural Sciences/National Agricultural Science Data Center, Beijing, China; 2 Sanya Nanfan Research Institute, Chinese Academy of Agricultural Sciences, Sanya, Hainan, China; 3 Institute of Crop Sciences, Chinese Academy of Agricultural Sciences, Beijing, China; 4 Rice Research Institute, Jiangxi Academy of Agricultural Sciences/Jiangxi Crop Germplasm Resources Research Center, Nanchang, China; 5 Biotechnology and Nuclear Technology Research Institute, Sichuan Academy of Agricultural Sciences Biotechnology and Nuclear Technology Research Institute, Sichuan Academy of Agricultural Sciences, Chengdu, China; 6 Heilongjiang Academy of Agricultural Sciences, Harbin, Heilongjiang, China

**Keywords:** deep learning, rice, phenotypic trait, panicle traits, precision extraction

## Abstract

**Introduction:**

Key rice panicle traits (grain number, panicle length, grain dimensions, maturity) determine yield and quality, and high-precision/high-throughput measurement is critical for rice breeding. Traditional methods are.

**Methods:**

A dataset of 5300 rice panicle images (loose/normal/dense types; milk/dough/full maturity/over-ripe stages) was constructed, with 3290 for training, 940 for validation, and 470 for testing. A deep learning pipeline integrating.

**Results:**

The panicle length extraction achieved R²=0.9583, RMSE=5.69 mm. Grain counting R² values were 0.9799 (loose), 0.9551 (normal), 0.9278 (dense). Grain length R²=0.8823, grain width MAPE=6.64%. OPG-YOLOv8.

**Discussion:**

This study provides a comprehensive, automated tool for rice panicle phenotyping, addressing occlusion challenges and bridging the gap between advanced models and breeding applications.

## 1 Introduction

Rice is widely cultivated globally (Tang et al., 2023), accounting for approximately 30% of the total grain planting area and serving as the staple food for over half of the world’s population (Yu et al., 2025). In China, rice is one of the most important food crops, occupying 30% of the national grain planting area and contributing 40% of the total grain yield (Zhang et al., 2024). Cultivating high-yield and high-quality rice varieties is an effective approach to addressing food security challenges (Ayumi et al., 2023). Rice panicle phenotypic traits are directly related to yield characteristics, making their acquisition a critical step in rice breeding research. These traits include panicle morphology and grain characteristics, such as panicle length, grain number per panicle, grain length, grain width, and maturity (Thesma et al., 2024). Therefore, rapid and accurate extraction of rice panicle phenotypic traits plays a pivotal role in rice breeding.

Traditional methods for measuring panicle traits, such as manual measurement with rulers and calipers, can evaluate rice panicle characteristics (Xiong, 2023). but these methods are labor-intensive, time-consuming, prone to grain damage, and susceptible to errors (Peng and Xie, 2020). In recent years, the integration of computer vision and artificial intelligence has emerged as a transformative solution in crop phenomics (Zhao et al., 2019; Yang et al., 2020). Specifically, deep learning has advanced high-throughput phenotyping technologies (Lu et al., 2023). Various deep learning methods, including seed counting, size determination, and panicle segmentation, have demonstrated impressive accuracy in crop trait detection (Wu et al., 2020). Similar deep learning-based approaches have also achieved success in other major crops, such as wheat ear counting (Pound et al., 2017; Ghosal et al., 2019). Current research efforts focus on extracting panicle traits from threshed rice panicles. For instance, Liu et al. (2017) developed a mobile application for rice and wheat grain counting with an error rate below 2%. Tan et al. (2019) proposed a method combining watershed and corner detection algorithms with neural network classification, achieving an average accuracy of 94.63%. However, the threshing process may damage rice grains, compromising result accuracy. Extracting traits from intact rice panicles is crucial. To address this, Gong et al. (2018) introduced a wavelet-based method to correct panicle area and edge contours, requiring manual panicle shaping and branch fixation for precise counting, with an average accuracy of 94%. Similarly, Lu et al. (2023) proposed a high-precision analysis method using visible light scanning and deep learning, achieving an R² of 0.99 between actual and detected grain counts. Despite these advancements, manual shaping remains time-consuming and costly, hindering large-scale phenotyping.

Recent studies have made progress in non-destructive extraction of rice panicle traits. For example, Sun et al. (2024b) proposed a high-throughput, non-destructive method (EOPT) for efficient and accurate panicle trait extraction, achieving an average grain counting accuracy of 93.57%, a mean absolute percentage error (MAPE) of 6.62%, and high precision in grain and panicle length measurements. Sun et al. (2024a) introduced an innovative method integrating object detection, image classification, and regression equations for accurate grain counting in natural morphology, with a counting accuracy of 92.60% and an MAPE of 7.69%. Wu et al. (2020) utilized transfer learning and Faster R-CNN for wheat grain counting across multiple scenarios, achieving an average accuracy of 91%. Misra et al. (Wu et al., 2020) introduced SpikeSegNet for wheat spike detection, reaching a counting accuracy of 95%. While existing studies have made significant progress in non-destructive rice panicle trait extraction, they still face certain challenges. Many methods focus on the extraction of a single trait, with insufficient comprehensive and simultaneous analysis of multiple key phenotypic traits. Additionally, most existing models remain at the algorithm level, lacking an end-to-end and automated integrated platform that covers the entire process from image acquisition to data analysis—and this limits their large-scale application in practical breeding work. Thus, the main challenge and contribution of this study lie in designing a general model capable of efficiently and accurately extracting multi-dimensional phenotypic traits, and encapsulating it in a user-friendly web-based system. This thereby provides one-stop technical support for breeding experts and truly advances the intelligent process of rice breeding.

Rice panicle structures vary significantly among cultivars, ranging from compact to loose morphologies (Misra et al., 2020). The primary challenge in rice panicle phenotyping lies in designing a universal model for efficient and accurate extraction of diverse panicle traits. In this study, we propose a deep learning-based method integrating object detection, dynamic DFS pruning, and linear regression. The panicle length extraction module combines image preprocessing with skeletonization and dynamic DFS pruning for length calculation. The grain counting module employs the OPG-YOLOv8 algorithm to enhance small object detection accuracy, incorporating panicle sparsity and regression models for grain number prediction. The grain length, width, and maturity extraction modules filter samples based on grain detection results, extract grain dimensions through a series of operations, and quantify yellowness to determine maturity. Finally, a web platform built on the Django framework and Python language provides an integrated service from data acquisition to cloud-based intelligent analysis, offering technical support for rice breeding and advancing intelligent and digitalized breeding processes.

## 2 Dataset construction and methods for extracting rice panicle phenotypic traits

### 2.1 Dataset construction

#### 2.1.1 Image data acquisition and processing

**Figure 1:**
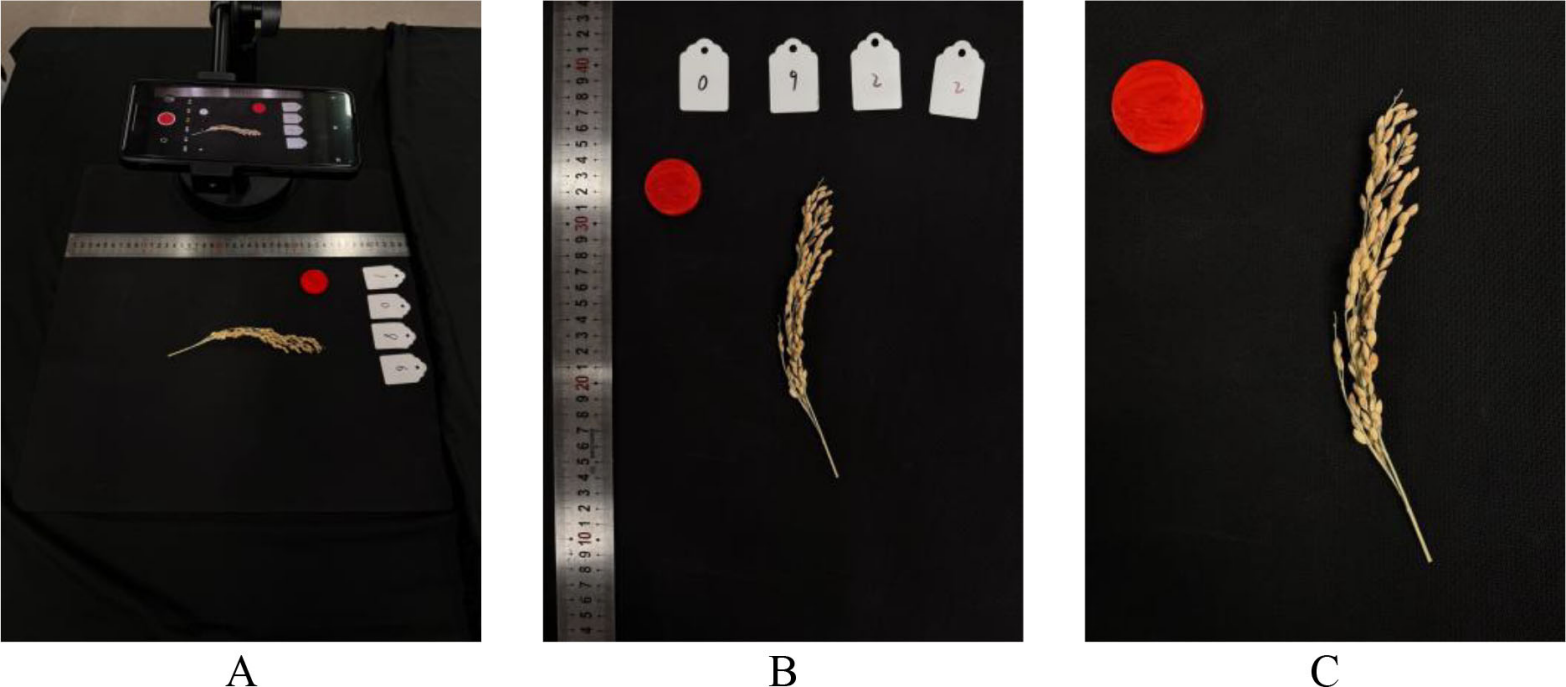
Rice panicle image acquisition workflow. **(A)** The imaging setup. **(B)** A raw image captured with the setup, featuring a red circular calibration board (30 mm in diameter) used as a size reference. **(C)** The final cropped panicle image used for trait extraction.

The rice samples used in this study were collected from the rice germplasm resources team at the Institute of Crop Sciences, Chinese Academy of Agricultural Sciences. Field data collection was conducted in September 2024 at the Yichun Gao’an Base in Nanchang, Jiangxi Province (28.3°N, 115.1°E), where phenotypic data were acquired from 1,772 rice germplasm accessions at maturity, including 1,338 japonica and 434 indica varieties, with a total of 4,700 panicle images collected. Supplementary validation data were collected from the Modern Agricultural Science and Technology Innovation Demonstration Park of Sichuan Academy of Agricultural Sciences (30.8°N, 104.2°E), an independent cross-region validation base—covering 200 local rice germplasm accessions (120 japonica and 80 indica varieties) with a total of 600 panicle images (exclusively for generalization verification, not involved in model training). Post-harvest panicle imaging was performed indoors under LED lighting (**Figure 1**). A smartphone mounted at a fixed distance of 30 cm from the samples captured images at a resolution of 3024×4032 pixels, with automatic ISO and shutter speed adjustment. Panicles were placed on a uniform black background alongside a 30 mm-diameter red calibration board to enable pixel-to-physical size conversion. A white numbered plate corresponding to field plot IDs ensured data consistency with planting records.

All captured images underwent a manual preliminary screening conducted by two trained researchers to ensure data quality. The screening criteria were to discard any image exhibiting: (1) significant motion blur where grain edges were not clearly visible; (2) poor focus across the majority of the panicle; (3) severe overexposure or underexposure that obscured grain details. This qualitative but standardized filtering step was crucial for removing unsuitable samples before further processing. Remaining images were cropped to isolate panicle regions and renumbered, yielding 4,700 high-quality images for subsequent processing.

#### 2.1.2 Manual measurement of rice panicle traits

**Figure 2:**
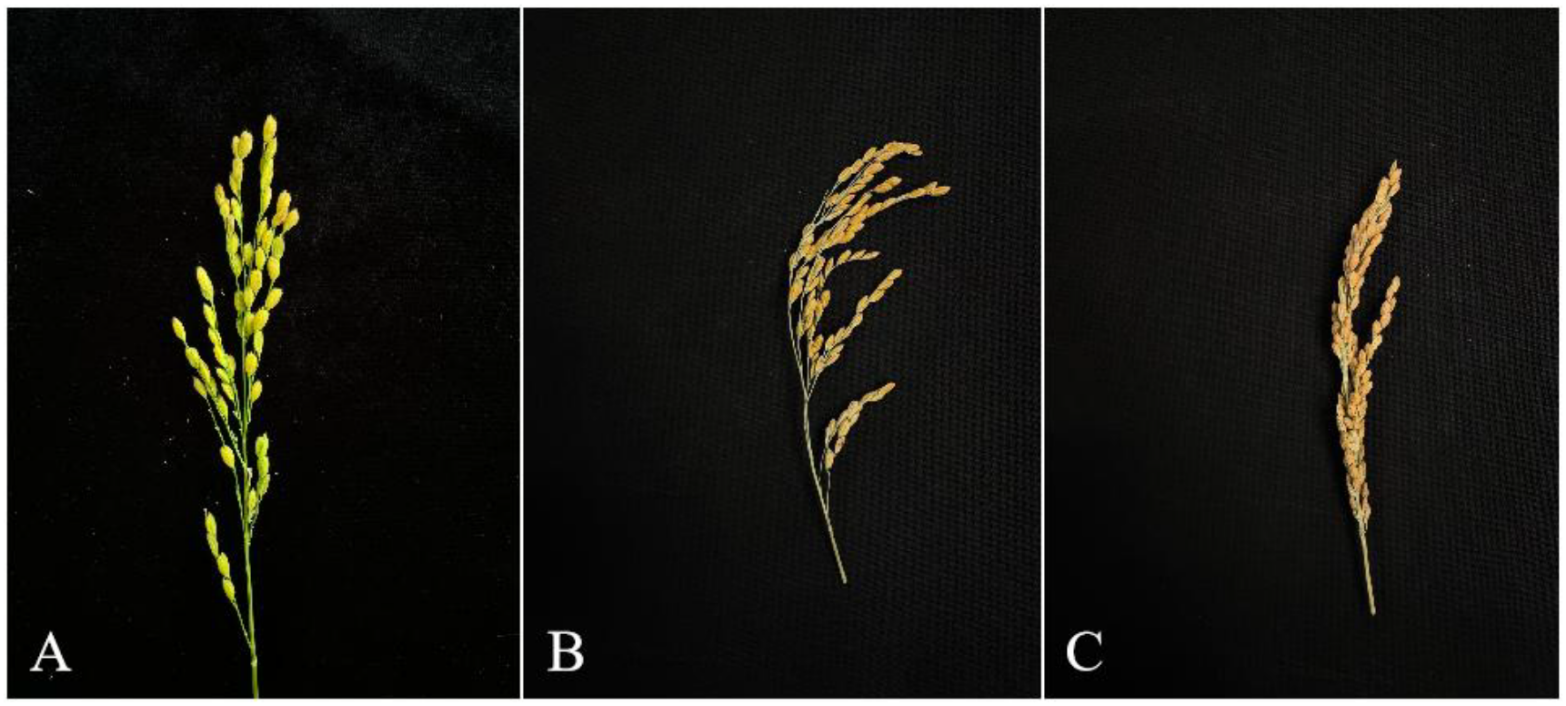
The three panicle types in this study. **(A)** Type 1 Panicles. **(B)** Type 2 Panicles. **(C)** Type 3 Panicles.

When using deep learning to obtain rice panicle traits, manually measured data of rice panicle traits are required to provide accurate real - world data for model training and testing. Therefore, when manually measuring rice panicle traits, we tried our best to keep the panicles intact, avoiding breaking or damaging them, and placed the panicles flat on the table. (1) Regarding the measurement of panicle length, 400 rice panicle samples were selected. A ruler with an accuracy of 1 mm was used to measure the length from the base to the top of the panicle parallel to the panicle to obtain the panicle length data. (2) For the counting of grains on the panicle, the number of grains on 600 rice panicles was manually counted. (3) When measuring the length and width of panicle grains, 240 rice panicle samples were selected, and a vernier caliper was used to measure the grains on the panicles. (4) In terms of evaluating the maturity of rice panicles, three rice breeding experts manually evaluated the maturity of rice panicle images to determine the maturity level of the panicles. (5) For the identification of rice panicle types, three rice breeding experts manually classified 3000 selected rice panicle images, with the classification criteria and typical panicle morphologies shown in **Figure 2**. The first type was “loose”, where the grains had slight mutual occlusion; the second type had the characteristic of “normal”, with a large amount of mutual occlusion between grains; the third type was “dense”, with severe mutual occlusion between grains, and the appearance was similar to a rod.

#### 2.1.3 Construction of the dataset of rice panicle traits

In this study, the 4,700 rice panicle images were partitioned according to their specific applications. A subset of images was annotated using LabelImg for distinct analytical purposes, with detailed allocations presented in **Table 1**. (1) A total of 1,000 images were designated for grain detection and performance evaluation across different models, specifically allocated as 700 for training the grain detection model, 200 for validation, and 100 for testing. (2) Among these, 3,000 images were utilized for panicle category detection, comprising 2,100 for training, 600 for validation, and 300 for testing. (3) From the 2,100 manually measured sample images, 400 were employed to validate the panicle length extraction algorithm, 660 for regression modeling, 240 for verifying the length-width extraction algorithm, and 1,200 for panicle grain maturity analysis.

**Table 1:** Details of dataset and images used in this study.

Dataset type	Sampled quantity (Images)	Specific usage and subdivision allocation	Notes
Original filtered image set	5300	High-quality rice panicle images obtained after deblurring and cropping, providing basic data for all sub-experiments	None
Panicle type classification sub-dataset	3000	For classifying rice panicle types (loose/normal/dense): 2100 images for training, 600 for validation, 300 for testing	Randomly sampled from the original filtered image set
Object detection and multi-model comparison sub-dataset	1000	Annotation for grain object detection (700 images for training, 200 for validation, 100 for testing); Multi-model performance comparison experiments	Randomly sampled from the original filtered image set; dual-purpose for two experiments
Phenotypic trait extraction validation sub-dataset	2100	Validation of panicle length extraction algorithm (400 images); Validation of grain length and width extraction (240 images); Grain maturity analysis (1200 images)	Randomly sampled from the original filtered image set; covers multi-trait validation
Independent cross-region validation dataset	600	Independent cross-region generalization verification	Only used for independent validation of trait extraction accuracy

### 2.2 Methods for extracting rice panicle phenotypic traits

Rice panicle phenotypic traits such as grain number per panicle, panicle length, grain length, and grain width are core indicators for evaluating rice yield and quality, and their accurate extraction is crucial for rice breeding selection. This section focuses on three key traits and proposes corresponding extraction methods, covering the entire process from image preprocessing to trait quantification, ensuring the efficiency and precision of extraction while maintaining compatibility with the constructed dataset.

**Figure 3:**
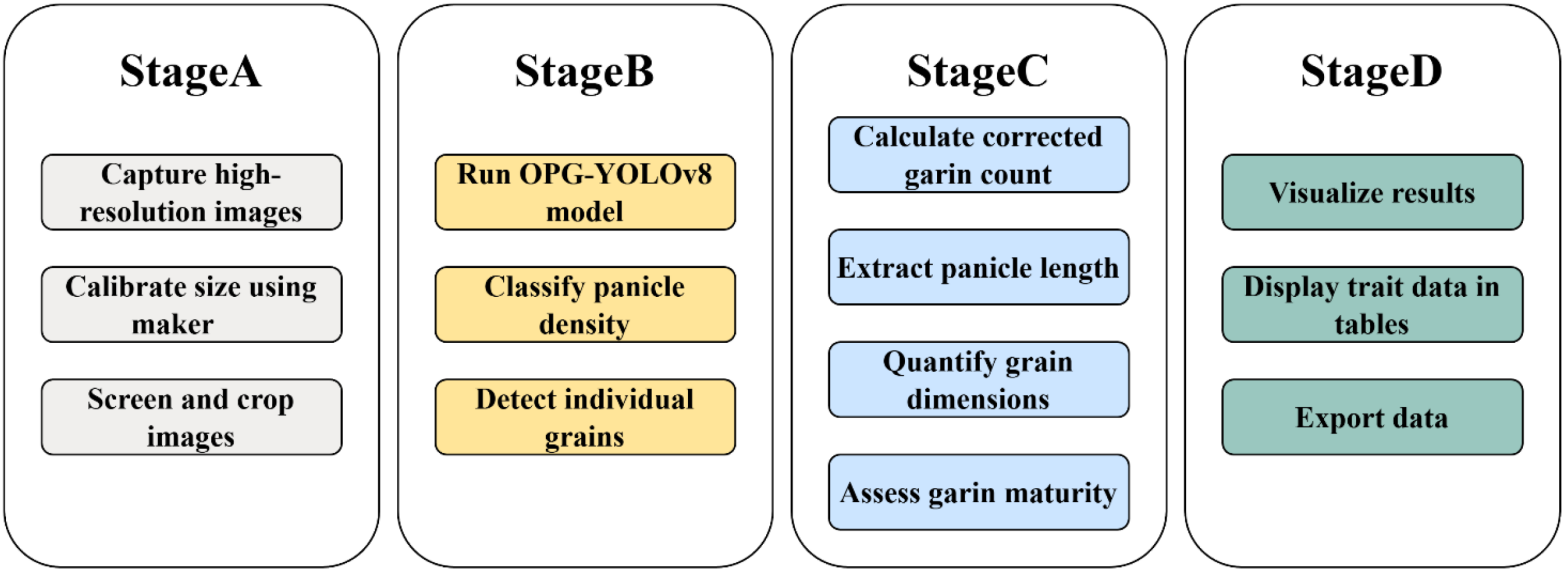
A high-level overview of the proposed workflow for rice panicle phenotypic trait extraction. The pipeline consists of four main stages: **(A)** Image Acquisition and Preprocessing, **(B)** AI-Powered Analysis, **(C)** Multi-Trait Quantification, and **(D)** Platform Interface and Data Export. This figure provides a conceptual map of the entire process.

**Figure 4:**
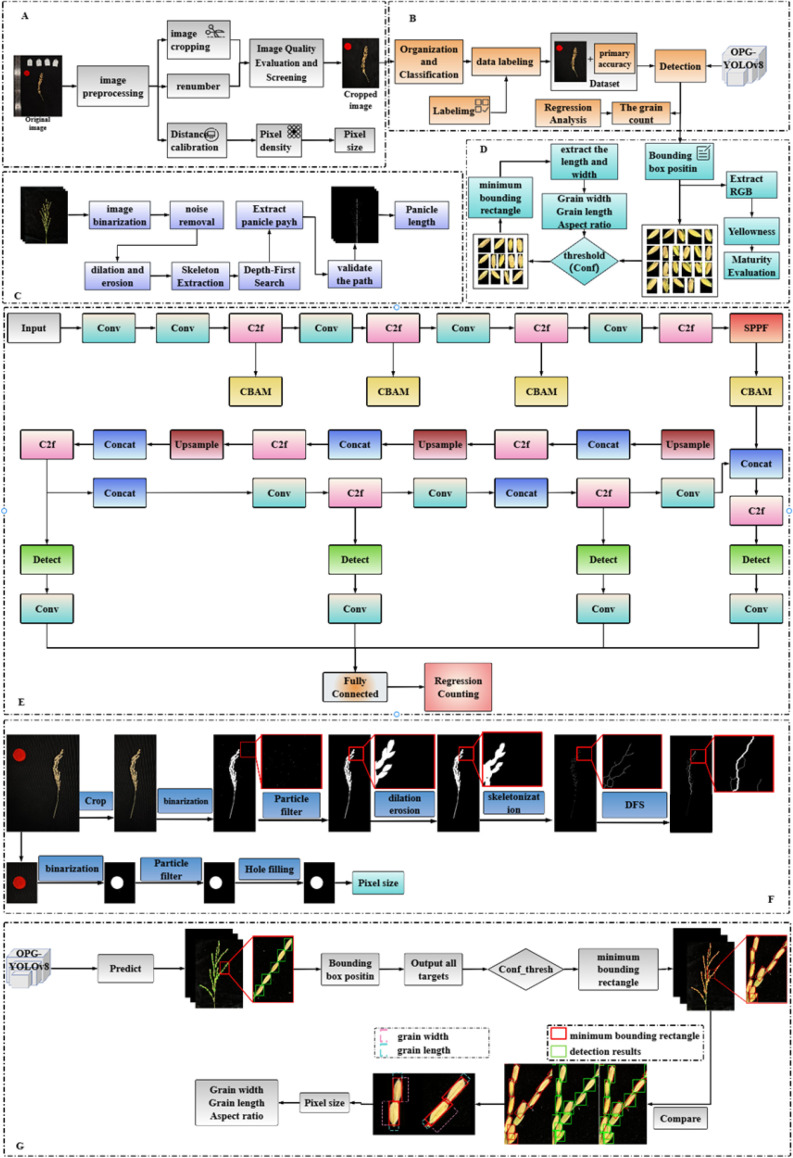
Detailed technical diagram of the trait extraction pipeline and model architecture. This figure provides a granular view of the algorithms and components referenced in **Figure 3**. **(A)** Image preprocessing and calibration steps. **(B)** The grain counting module. **(C)** The panicle length extraction module. **(D)** The grain dimension and maturity extraction module. **(E)** The detailed architecture of our modified OPG-YOLOv8 network, highlighting the integration of CBAM modules. **(F)** Algorithmic flowchart for panicle length extraction. **(G)** Algorithmic flowchart for grain length and width extraction.

To accurately and automatically obtain key phenotypic traits from rice panicles, we developed a comprehensive deep learning-based pipeline. A high-level overview of this four-stage workflow is presented in **Figure 3**, illustrating the process from image acquisition to final data output. The underlying technical implementation, including the specific algorithms and the detailed architecture of our proposed OPG-YOLOv8 network, is detailed in **Figure 4**. Our model is composed of four primary modules: (1) an image pre-processing and calibration module; (2) a grain counting module; (3) a panicle length extraction module; and (4) a module for extracting grain length, width, and maturity. The specific methods employed within each module are described in the following sections.

#### 2.2.1 The OPG-YOLOv8 model for grain detection

Accurate detection of individual rice grains is the foundation for multiple trait extraction tasks, including grain counting, dimension measurement, and maturity analysis. To achieve this with high accuracy and speed, we proposed an improved OPG-YOLOv8 detection algorithm, with its architecture detailed in **Figure 4E**. This algorithm enhances the standard YOLOv8 model by primarily optimizing its neck and head structures (Jocher et al., 2023).

Specifically, we introduced the CBAM (Convolutional Block Attention Module) to suppress background noise in shallow-layer features (Woo et al., 2018). The CBAM is an attention mechanism composed of a channel attention module (CAM) and a spatial attention module (SAM). By selectively weighting the channel and spatial information, it significantly enhances the feature representation ability for small targets like rice grains. Furthermore, a higher-resolution feature map is introduced in the neck for multi-scale feature fusion to compensate for information loss during feature transmission. Concurrently, an additional detection head is added to the head of OPG-YOLOv8, which utilizes the high-resolution feature map for prediction, effectively improving the detection accuracy of small targets. This optimized model serves as the core engine for all subsequent grain-level phenotypic analyses.

#### 2.2.2 A two-stage method for accurate grain counting via density classification and regression

Under natural conditions, the mutual occlusion of grains within a panicle poses a significant challenge to accurate counting based solely on object detection. Denser panicles can have a substantial number of grains hidden from view, leading to systematic underestimation. To address this challenge, we developed a two-stage method that integrates density classification with regression modeling to correct the initial count.

The first stage involves classifying the overall panicle morphology to estimate the potential level of grain occlusion. Based on expert evaluation, we defined three density categories reflecting the degree of mutual grain occlusion: “loose”(slight occlusion),”normal”(moderate occlusion), and” dense”(severe occlusion), with typical examples shown in **Figure 2**. The OPG-YOLOv8 model was trained on a dedicated dataset to automatically recognize the visual patterns of these types and assign an input panicle image to the most appropriate category. This classification provides the crucial contextual information needed for the subsequent correction step.

The OPG-YOLOv8 model was trained on a dedicated dataset to automatically recognize and assign an input panicle image to one of these three categories.

In the second stage, the classification result from Stage 1 is used to apply a targeted correction to an initial grain count. First, the OPG-YOLOv8 object detection model is used to obtain a preliminary predicted count of all visible grains in the panicle (denoted as $X_{i}$). Then, based on the panicle’s assigned density category, the corresponding pre-established linear regression equation is selected to calculate the final, corrected grain count (denoted as $Y_{i}$).

This regression model was built to empirically correct for the systematic underestimation of grain counts caused by occlusion in denser panicles (Equations 1–3). The derivation and validation process was as follows: a dedicated regression modeling sub-dataset of 660 panicle images (220 for each density category) with manually verified, ground-truth grain counts was utilized. First, the OPG-YOLOv8 object detection model was applied to this dataset to obtain the initial predicted count of visible grains (denoted as X_i_) for each image.

Subsequently, for each density category, a simple linear regression analysis was performed by fitting these predicted counts (X_i_) against their corresponding manual ground-truth counts using the least-squares method. The resulting equations represent the best-fit linear models that map the visible grain count to the estimated total grain count for each respective category. The equations for the three density classes (1-loose, 2-normal, 3-dense) are presented as follows:

(1) $$Y_{1}=1.136X_{1}+12.67$$

(2) $$Y_{2}=1.555X_{3}-6.191$$

(3) $$Y_{3}=1.821X_{3}-29.2$$

**Figure 5:**
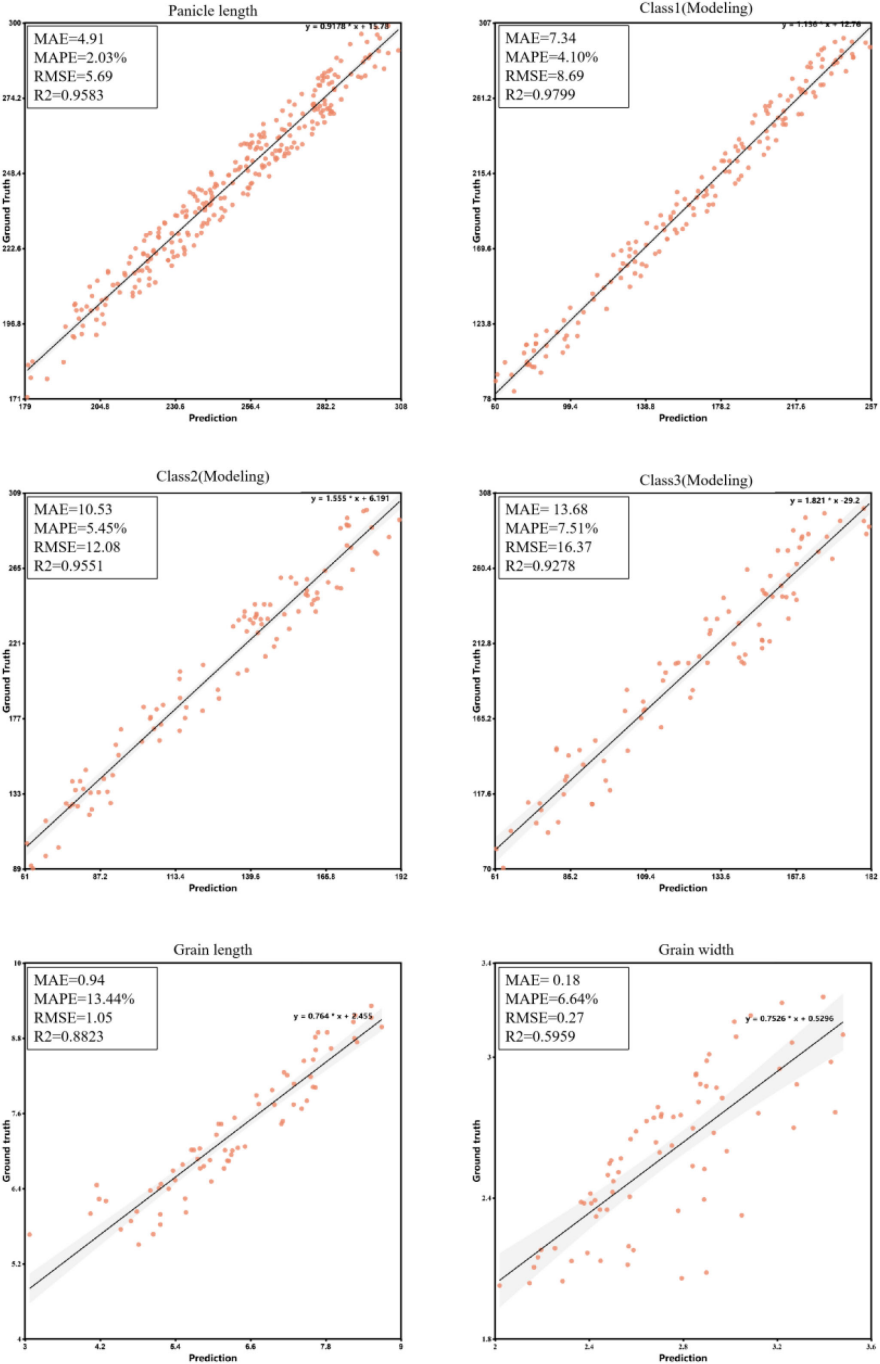
Validate the extraction results of rice panicle phenotypic traits.

In this framework, X_i_ represents the initial predicted grain count from the object detection model for a panicle in category *i*, while Y_i_ represents the final, more accurate corrected grain count. The validation of these models is demonstrated by the high coefficient of determination (R²) values presented in the Results section (**Figure 5**), which confirm a strong goodness of fit and a significant linear relationship between the predicted and true values. This two-stage approach allows the system to systematically compensate for hidden grains based on the panicle’s structure, significantly improving the overall counting accuracy.

#### 2.2.3 Method for extracting panicle length

To accurately measure the panicle length, this study proposed a panicle length extraction algorithm based on the dynamic pruning strategy of the depth-first search (DFS) algorithm. The calculation process of this method is shown in **Figure 4F**. First, the image is grayscaled, and then it is filtered to remove noise, eliminating interferences caused by the variability of lighting conditions and the physical limitations of the camera sensor. Subsequently, threshold segmentation is performed to separate the panicle part from the background. Finally, morphological closing operations are carried out to fill holes and connect edges, prominently highlighting the main characteristics of the panicle.

The skeletonization function in the skimage library (Wu et al., 2019) is used to extract the central axis of the panicle. To adhere to standard agronomic measurement practices, the panicle length was defined as the distance from the panicle neck to the furthest tip of the panicle. In our algorithm, the panicle neck is automatically identified as the lowest branching point on the extracted skeleton. This ensures that the main panicle stalk below this point is consistently excluded from the length calculation.

Following skeletonization, the DFS pruning algorithm (Song et al., 2019) is employed for path planning to depict the main path of the panicle. The dynamic pruning strategy of the DFS algorithm constructs a two-dimensional matrix from the pre-processed panicle image. One end of the skeleton is selected as the starting point, and the DFS algorithm is used to traverse the graph structure of the panicle (Yao et al., 2017). During the search process, the length of the current path is recorded. When a new node is searched, the length of the current path is compared with the recorded optimal length. If the length of the current path is less than the optimal length, the search continues; if it is greater than or equal to the optimal length, pruning is performed, and the search for this path is no longer continued. According to the recorded path information, the main path of the panicle can be depicted on the original image or a new image, and then the actual panicle length is calculated based on the pixel-to-physical size conversion factor (Hu et al., 2016).

#### 2.2.4 Method for extracting grain length and width

Based on the position information of the grains in the panicle detected by the OPG - YOLOv8 model proposed in this study, the bounding box information of the rice panicle grains is determined according to the detection results, and then the length and width are extracted. However, due to the occlusion of grains in the panicle, it may happen that the detection box does not completely contain the rice panicle grains. Therefore, the detection results need to be screened. When screening the grains, Conf (confidence threshold) is used to measure the confidence level of the model’s prediction of the detected target. Its value range is usually between 0 and 1. The higher the value, the more confident the model is about the accuracy of the detection result. Conf is used to determine whether the prediction box accurately covers the target grain. Only the boxes that exceed the set threshold are considered valid samples. After repeated screening and verification in this experiment, the rice panicle grains with a confidence threshold greater than 0.8 are further measured for length and width. After segmenting the selected grains, the length and width of the grains are extracted. The extraction process is shown in **Figure 4G**. It is important to note that the initial object detection model provides a bounding box primarily for locating each grain, which is not used for direct measurement. This is because initial bounding boxes can sometimes fail to capture the entire grain, particularly slender tips or awns, due to occlusion or morphological variations, which could introduce measurement inaccuracies.

To ensure high-precision measurement, our method employs a more robust two-step process. First, the coordinates of the initial bounding box are used to isolate a region of interest (ROI) containing the target grain. Second, within this ROI, we perform a precise threshold segmentation and particle filtering to extract the exact pixel morphology of the grain body. Finally, the minimum bounding rectangle is calculated based on this accurate segmentation mask, not the initial detection box. This segmentation-based approach ensures that the final length and width measurements accurately reflect the true dimensions of the grain by systematically overcoming potential inaccuracies in the initial detection phase.

#### 2.2.5 Method for extracting panicle grain maturity

To objectively quantify grain maturity, a method based on the color characteristics of the panicle was developed. While the RGB color space from digital images captures fundamental color information, it is not perceptually uniform, meaning the numerical distance between two RGB values does not directly correspond to the human-perceived color difference. To establish a more robust and human-centric metric, a multi-step workflow was designed to convert the initial RGB data into the Hunter Lab color space, which is approximately uniform for human color perception. This ensures that the final calculated yellowness index (YI) provides a consistent and meaningful measure of maturity.

The yellowness extraction for grains leverages the detection results from the OPG-YOLOv8 model. For each detected grain, the calculation process begins with its average RGB values normalized via Equation 4, and proceeds as follows:

(4) $$\begin{array}{c} r=\frac{R}{255}\\ g=\frac{G}{255}\\ b=\frac{B}{255} \end{array}$$

Since the human eye’s perception of color is nonlinear, and the RGB color space is a linear space based on physical devices, in order to more accurately simulate the visual characteristics of the human eye, gamma correction is required and implemented via Equation 5. The specific formula is as follows:

(5) $$\text{If r}>0.04045\;,\text{then r}=(\left(\text{r}0.055\right)/1.055{)}^{2.4}\;,\text{else r}=\text{r}/12.92$$

(the same approach applies to g and b)

After gamma correction, the normalized and corrected RGB values are converted to CIE XY values using the following formula (Equation 6):

(6) $$\begin{array}{c} \text{X}=0.4124*\text{r}+0.3576*\text{g}+0.1805*\text{b}\\ \text{Y}=0.2126*\text{r}+0.7152*\text{g}+0.0722*\text{b}\\ \text{Z}=0.0193*\text{r}+0.1192*\text{g}+0.9505*\text{b} \end{array}$$

In the CIE XYZ color space, X, Y, and Z represent distinct color components. Based on the obtained CIE XYZ values, the Hunter Lab parameters are calculated. In the Hunter Lab color space, L denotes Lightness, which reflects the brightness of the color calculated by Equation 7; a represents Chromaticity in the red-green axis, indicating the color’s shift along the red-green direction; and b signifies Chromaticity in the yellow-blue axis, representing the color’s shift along the yellow-blue direction. The formulas for calculating these parameters are as follows:

(7) $$\begin{array}{c} \text{a}=\left(175*\left(\text{X}-\text{Y}\right)\right)/\left(\sqrt{\text{Y}}\right)\\ \text{b}=\left(70*\left(\text{Y}-\text{Z}\right)\right)/\left(\sqrt{\text{Y}}\right)\\ \text{L}=10\sqrt{\text{Y}} \end{array}$$

These transformations from the RGB color space to the Hunter Lab space follow the standard colorimetric principles and formulas established by the International Commission on Illumination (CIE) (CIE, 2004). Finally, the yellowness value is calculated based on the aforementioned data results, using the following formula:

(8) YI=(128×L+100×a+30×b)/100

Through this formula Equation 8, the information of lightness (L) and chromaticity (a, b) is integrated, enabling the precise quantitative calculation of yellowness. This yields a numerical value that accurately reflects the degree of yellow in the color.

Based on the comparison between the rice maturity results judged by rice breeding experts and the average yellowness values, this study established a clear standard for the classification of rice maturity stages, as presented in **Table 2**:

**Table 2:** Criteria for the classification of rice maturity stages.

Growth stage of rice	Range of average yellowness value
Milk Stage	Less than 50
Dough Stage	50 - 75
Full Maturity Stage	75 - 100
Over-ripe Stage	Greater than 100

### 2.3 Experimental setup and training details

All deep learning training was conducted on a Windows operating system equipped with an 8-core Intel(R) Core i9-11900K CPU (3.50 GHz) and an NVIDIA GeForce RTX 3080 GPU. The CUDA version used was 12.6. The software used for model training was PyCharm 2023.3 Community Edition (JetBrains, Czech Republic), and Origin 2024 (OriginLab Corporation, USA) was used for fitting regression equations. The specific training hyperparameters for each compared model are detailed in **Table 3**.

**Table 3:** Training parameters for different models.

Hyperparameters	OPG-YOLOv8	YOLOv8	YOLOv7	Mask R-CNN	EfficientDet
batch_size	8	8	8	2	32
momentum	0.937	0.937	0.937	0.9	0.93
learning rate	0.01	0.01	0.01	0.001	0.002
optimizer	SGD	SGD	SGD	SGD	SGD
weight_decay	0.0005	0.0005	0.0005	0.0005	0.0001

### 2.4 Performance evaluation metrics

The performance of the proposed models was assessed using a set of standard evaluation metrics, tailored for object detection and regression tasks.

For the grain detection models, performance was evaluated based on mean Average Precision (mAP), Parameters (Params), Floating-point Operations (FLOPs), and Frames Per Second (FPS). The mAP, a standard metric for evaluating the accuracy of object detectors, was calculated at an Intersection over Union (IoU) threshold of 0.5 (mAP50) (Everingham et al., 2010). Params and FLOPs were used to measure model complexity and computational cost, while FPS was used to evaluate inference speed. For the regression tasks, which include panicle length, grain dimension, and grain count estimations, the following metrics were used to measure the correlation and error between predicted values and ground-truth values (Botchkarev, 2018):

- Coefficient of Determination (R²): Measures how well the predicted values fit the actual values. A value closer to 1 indicates a better fit.
- Mean Absolute Error (MAE): Measures the average absolute difference between predicted and actual values.
- Mean Absolute Percentage Error (MAPE): Expresses the MAE as a percentage of the actual values, useful for understanding the error in relative terms.
- Root Mean Square Error (RMSE): Represents the standard deviation of the prediction errors.

The mathematical formulas for these metrics are defined as follows (Equations 9–15):

(9) $$MAPE=\left(1/n\right)*\sum \left(\left|\left(N_{actual\;}-N_{predict\;}\right)/N_{actual\;}\right|\right)*100\%$$

(10) $$Params\;=C_{in\;}^{*}C_{out}^{*}K_{h}^{*}K_{w}+C_{out\;}$$

(11) $$FLOPs=\;Params\;*M_{outh\;}^{*}M_{ounw\;}$$

(12) $$mAP=\sum_{i=1}^{c}APi/C$$

(13) $$RMSE=\sqrt{\left(\left(\sum {\left(N_{actual\;}-N_{predict\;}\right)}^{2}\right)/n\right)}$$

(14) $$MAE=\left(1/n\right)*\sum \left(\left|N_{actual\;}-N_{predict\;}\right|\right)$$

(15) $$R^{2}=1-\sum \left({\left(N_{actual\;}-N_{predict\;}\right)}^{2}\right)/\sum \left({\left(N_{actual\;}-\langle N_{actual\;}\rangle \right)}^{2}\right)$$

Among these evaluation indices, $C_{in\;}$ represents the number of input channels, $C_{out\;}$ represents the number of output channels, $K_{h\;\;},K_{w}$ represents the size of the convolutional kernel, $M_{outh},M_{ounw\;}$ represents the height and width of the output feature map, C represents the total number of categories, APi represents the AP value of the i-th category, n represents the number of samples, $N_{actual\;}$ represents the true value, which may be the true number of grains. $N_{predict\;}$ represents the predicted value, that is, the number of grains in the rice panicle detected by the object detection model.

## 3 Experimental results and analysis

### 3.1 Evaluation of the extraction results of rice panicle phenotypic traits

The performance of the OPG-YOLOv8 model in predicting rice panicle types is presented in **Table 4** Among them, there are 213 samples of Class 1, with 195 correctly predicted, and the accuracy rate is 91.55%; there are 178 samples of Class 2, with 154 correctly predicted, and the accuracy rate is 86.52%; there are 247 actual samples of Class 3, with 238 correctly predicted, and the accuracy rate is as high as 96.36%. The model has the best prediction effect for Class 3 rice panicles, while the prediction accuracy of Class 2 is relatively low, possibly because the characteristics of this type of rice panicle are easily confused with those of other categories.

**Table 4:** Prediction results of different types of rice panicles.

Panicle types	Number of true samples	Number of correctly predicted samples	Accuracy
Class 1	213	195	91.55%
Class 2	178	154	86.52%
Class 3	247	238	96.36%

The accuracy and robustness of the panicle length extraction algorithm were evaluated. The results showed a strong correlation between predicted and actual lengths, with an R² of 0.9583, an RMSE of 5.69 mm, an MAE of 4.91 mm, and a MAPE of 2.03%.

The performance of the two-stage grain counting method was evaluated across the three panicle density categories, and the results are detailed in **Figure 5**. The model demonstrated a strong linear relationship between predicted and true values for all types. For the ‘loose’ panicles (Class 1), the model achieved the highest accuracy with an R² of 0.9799 and an RMSE of 8.69. As panicle density increased, the R² values remained high for ‘normal’ (0.9551) and ‘dense’ (0.9278) categories, confirming the method’s effectiveness.

However, a corresponding increase in error metrics was observed with density, with the RMSE reaching 16.37 for the ‘dense’ category. This trend highlights the inherent challenge of severe grain occlusion in denser panicles, a factor that increases prediction variance. Despite this, the Mean Absolute Percentage Error (MAPE) for the ‘dense’ category remained low at 7.51%, indicating that the model maintains a high level of relative accuracy even in the most challenging cases.

### 3.2 Cross-region generalization verification results

To evaluate the generalization capability and robustness of our proposed model across different geographical regions and genetic backgrounds, we conducted tests on an independent, cross-region validation dataset. This dataset comprises 600 panicle images from the Modern Agricultural Science and Technology Innovation Demonstration Park of Sichuan Academy of Agricultural Sciences, which were not used in any part of the model training process. The performance of the model on the key phenotypic traits is summarized and compared with the original validation set in **Table 5**.

**Table 5:** Performance comparison of the model on the original and cross-region validation datasets.

Trait category	Metric	Original validation set	Cross-region validation set
Panicle length	R²	0.9583	0.9412
Grain count	RMSE (mm)	5.69	6.15
	Average R²	0.9543	0.9255
	Average MAE	10.52	12.88
Grain length	R²	0.8823	0.8655
Grain width	R²	0.5959	0.5731
Maturity stage	Classification Accuracy (%)	92.0%	89.5%

The results demonstrate that the model maintains strong performance and high accuracy on this entirely new dataset. For panicle length extraction, the R² value reached 0.9412, showing only a marginal decrease compared to the original validation set (0.9583) and indicating the high robustness of the measurement algorithm. In the critical task of grain counting, the model achieved an average R² of 0.9255, confirming the effectiveness of the two-stage counting method in handling novel panicle morphologies. Similarly, metrics for grain dimension extraction and maturity classification remained at a high level, with grain length R² at 0.8655 and maturity classification accuracy at 89.5%.

While there was an expected, slight decline in performance across all metrics, this can be attributed to subtle variations in panicle morphology, imaging conditions, or genetic backgrounds characteristic of the new region. Overall, these results strongly validate that our phenotyping model possesses excellent generalization ability, making it a reliable and applicable tool for broader practical breeding applications beyond the initial training environment.

### 3.3 Comparative experiment of grain detection models

**Table 6** presents the evaluation results of different models. In terms of detection accuracy, OPG-YOLOv8 demonstrates outstanding performance, achieving a mAP50 of 99.10%, which surpasses models such as YOLOv8 and YOLOv7. This indicates that OPG-YOLOv8 excels in target recognition and localization precision at an IoU threshold of 0.5. Regarding the R² metric, although OPG-YOLOv8’s score of 0.8 is slightly lower than YOLOv7’s 0.86, it still reflects strong data-fitting capability, comparable to YOLOv8’s 0.84 and superior to Mask R-CNN and EfficientDet. In terms of error metrics, OPG-YOLOv8 achieves an MAE of 57.4, which, although higher than YOLOv7’s 48.1, remains within an acceptable range for practical applications. Moreover, its overall accuracy advantage mitigates the relative impact of this metric to some extent. The MAPE of 30.47% is higher than that of other comparative models. The RMSE of 69, while relatively high, indicates a greater dispersion of prediction errors; however, combined with its high precision, it remains within acceptable limits. From the perspective of model complexity, OPG-YOLOv8 has 59.6M parameters and 113.2G FLOPs, demonstrating relatively low computational complexity and offering advantages in hardware resource utilization. The inference speed of OPG-YOLOv8 reaches 154 FPS, significantly higher than that of the other models. Overall, OPG-YOLOv8 exhibits superior comprehensive performance in object detection tasks, achieving an excellent balance between accuracy, computational complexity, and inference speed, and holds a clear advantage over other models.

**Table 6:** Comparison of different object detection networks on the validation set.

Models	mAP50	R^2^	MAE	MAPE	RMSE	Params	FLOPs	FPS
OPG-YOLOv8	99.10%	0.8	57.4	30.47%	69	59.6M	113.2G	154
YOLOV8	97.40%	0.84	52.2	28.41%	64	68.7M	180.3G	133
YOLOv7	96.10%	0.86	48.1	25.27%	62	53.9M	213.6G	87
Mask R-CNN	76.04%	0.81	54.8	27.86%	72	43.2M	247.5G	43
EfficientDet	84.32%	0.74	42.8	24.71%	58	37.4M	289.1G	35

### 3.4 Evaluation experiment of the maturity of panicle grains

**Figure 6:**
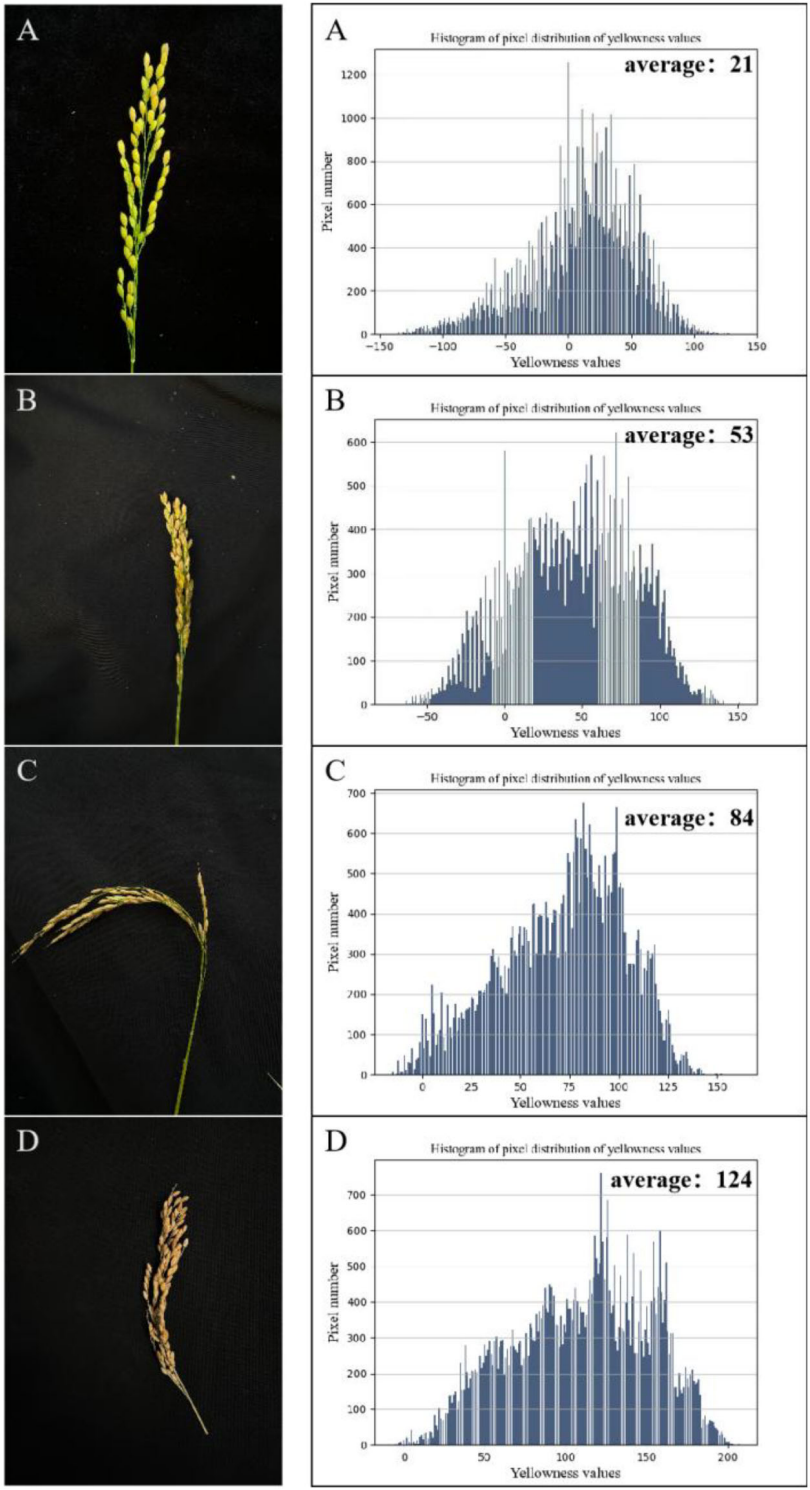
Histogram of grain maturity statistics for rice panicles. **(A)** rice panicles at the milk-ripe stage. **(B)** rice panicles at the dough stage. **(C)** Full-ripe rice panicles. **(D)** Overripe rice panicles.

The maturity of rice grains was assessed based on the mean yellowness values of panicles. A total of 1,200 panicle images were selected as experimental samples, and the average yellowness was statistically analyzed for each image. As shown in **Figure 6**, representative panicle images and their corresponding yellowness histograms are presented for four maturity stages: over-ripe, fully ripe, wax-ripe, and milk-ripe. The figure demonstrates marked variations in both the yellowness histograms and mean values across different maturity stages, confirming the discriminative capacity of yellowness metrics in characterizing grain maturation progression.

**Figure 7:**
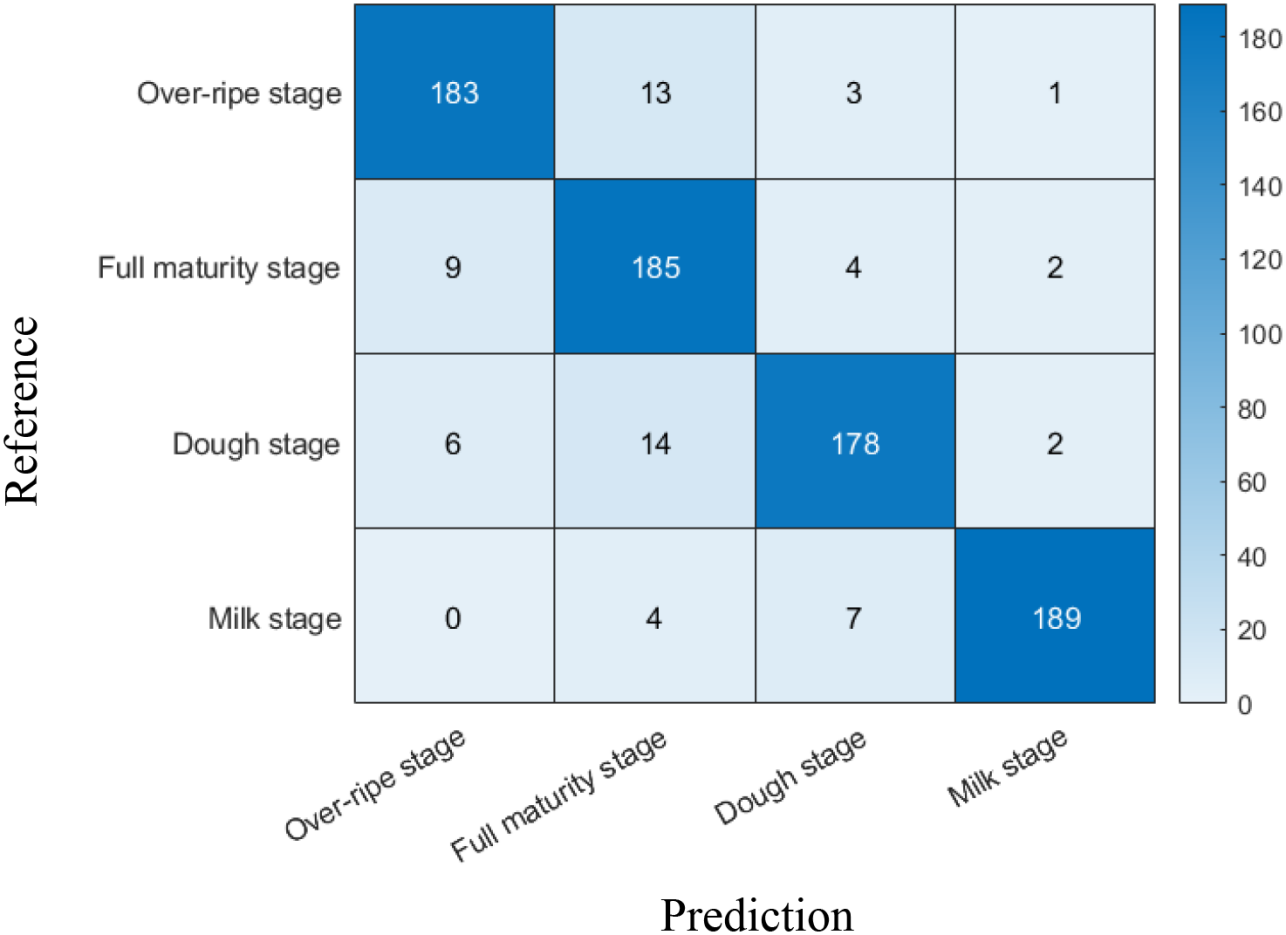
Confusion matrix for predicting the maturity of rice panicles.

The model’s performance in classifying rice panicle maturity stages (over-ripe, fully ripe, wax-ripe, and milk-ripe) was evaluated using a confusion matrix. As shown in **Figure 7**, the model’s maturity prediction was evaluated using 200 images per stage (totaling 800 samples) randomly selected from a dataset of 1,200 expert-annotated panicle images. The confusion matrix analysis revealed distinct classification accuracies across stages: over-ripe samples achieved 183 correct predictions (91.5% accuracy), with 13 misclassified as fully ripe; fully ripe panicles yielded 185 correct identifications (92.5% accuracy), though 9 were erroneously labeled as over-ripe; wax-ripe panicles attained 178 correct classifications (89.0% accuracy), with 6 and 14 misclassifications to over-ripe and fully ripe stages, respectively; while milk-ripe panicles demonstrated the highest accuracy (189 correct predictions, 94.5%), with only 4 and 7 misclassifications to fully ripe and wax-ripe stages. The diagonal values (correct classification rates) highlight the model’s capability to discern maturity phases, particularly excelling in milk-ripe stage recognition. Notably, inter-stage confusion predominantly occurred between adjacent maturity phases (e.g., over-ripe vs. fully ripe), suggesting challenges in distinguishing subtle transitional features. These results validate the model’s effectiveness in maturity stage identification while underscoring potential optimization avenues for boundary case refinement.

### 3.5 Research and development of a precision extraction system for rice panicle traits

**Figure 8:**
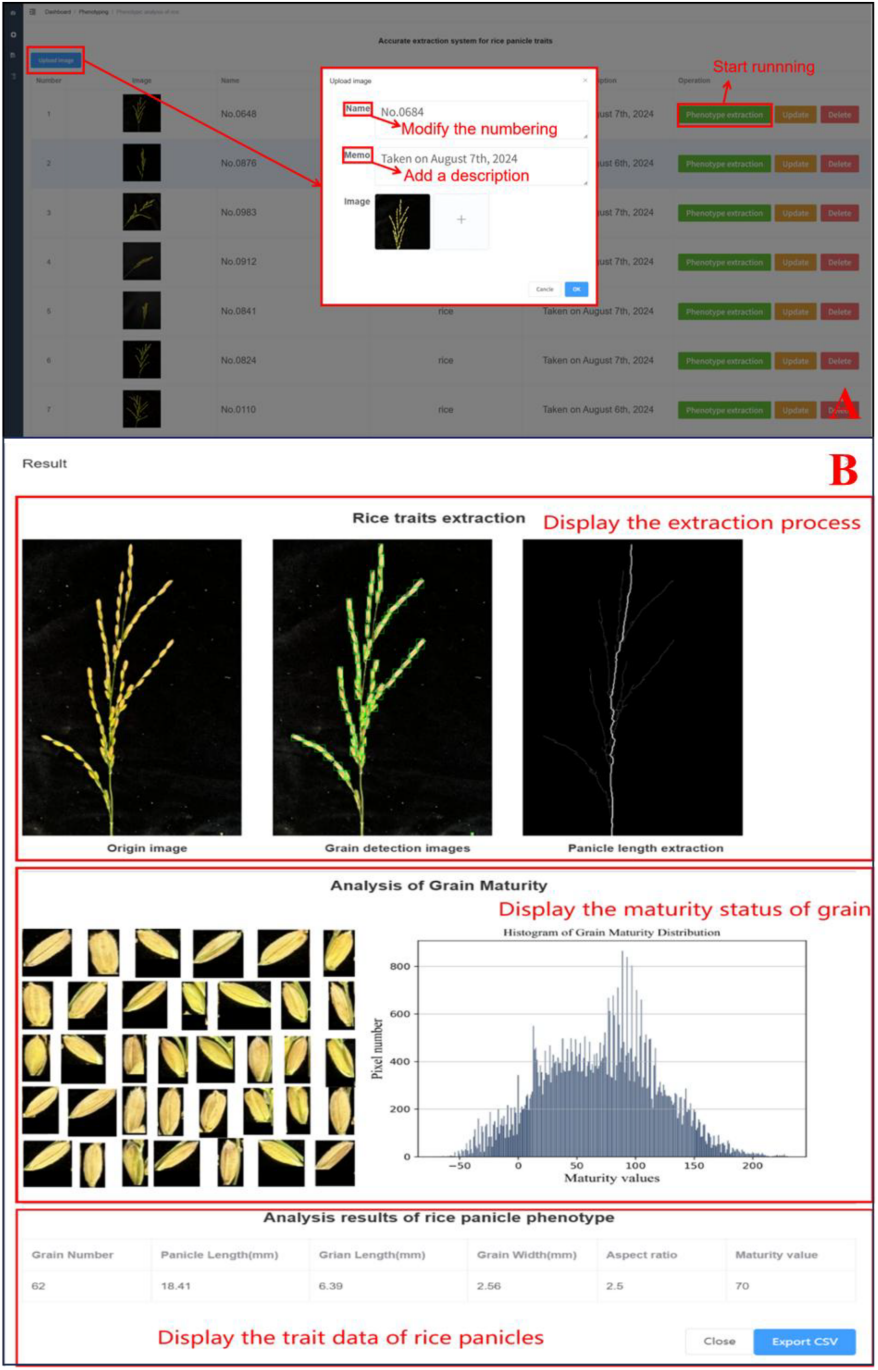
The user interface of the rice panicle trait extraction system. **(A)** The main interface for image upload and batch processing. **(B)** The results interface displaying the multi-trait extraction outputs.

To enable the practical application of the OPG-YOLOv8 detection model and panicle trait extraction algorithms, a “Rice Panicle Trait Extraction System” (as shown in **Figure 8**) was developed based on the Django framework and Python. The system incorporates a web-based interface module that supports multi-user, multi-role remote operations and data management. It provides an integrated workflow from data acquisition to cloud-based intelligent analysis, processing each image within an average of 5 seconds. The system rapidly and accurately extracts key phenotypic traits, including panicle length, grain length, grain width, length-to-width ratio, and maturity stage. Equipped with data-driven phenotyping algorithms, this platform delivers precise and efficient technical support for modern agricultural breeding research, bridging the gap between advanced computer vision models and real-world agronomic applications. he system is currently deployed as an internal web-based platform. To facilitate broader academic use, a demonstration version or open-access availability is under consideration for future development. The source code for the system’s core algorithms is included in the project’s GitHub repository, as mentioned in the Data Availability Statement.

## 4 Discussion

In this study, we developed and validated a deep learning-based system for the automated, high-throughput extraction of multiple, agronomically important phenotypic traits in rice panicles. The discussion below contextualizes our key findings, addresses their practical implications for rice breeding, and outlines the limitations and future directions of this work.

### 4.1 Accuracy and implications of panicle and grain trait extraction

Our system demonstrated high precision in measuring panicle length (R² = 0.9583), a key architectural trait often positively correlated with the total number of grains a panicle can hold. The robustness of our image pre-processing workflow combined with a dynamic DFS pruning strategy proved highly effective. This automated and accurate measurement provides rice breeders with a reliable tool for rapidly selecting genotypes with high-yield potential.

A cornerstone of our study is the novel two-stage method for grain counting, which integrates object detection with density-based regression. This approach effectively mitigates the common issue of underestimation caused by severe grain occlusion, a significant limitation in previous studies. The ability to rapidly and accurately screen thousands of genetic lines for grain number per panicle—a primary component of final yield—can dramatically accelerate the selection of elite genotypes. While automated panicle type recognition greatly enhances efficiency, the universality of this classification model could be further improved by incorporating a more diverse training set, including panicles from a wider range of genetic backgrounds and environmental conditions.

The system also provides valuable data on grain dimensions and maturity, which are fundamental to rice quality. While the extraction of grain length was satisfactory, accurately measuring grain width proved more challenging (R² = 0.5959). This limitation likely stems from the inherent difficulty of representing a complex 3D grain shape using a 2D minimum bounding rectangle. Future research could explore multi-view imaging or 3D reconstruction techniques to capture grain plumpness more accurately. Nevertheless, the automated measurement of length and width is highly valuable, as these traits collectively determine grain shape and size, which are critical quality characteristics influencing milling yield and consumer preference. Similarly, our objective maturity assessment via a quantified yellowness index offers breeders a powerful, data-driven tool to select for uniform ripening and optimize harvest timing, thereby maximizing both grain quality and yield.

### 4.2 Performance and limitations of the OPG-YOLOv8 model

The OPG-YOLOv8 model, which forms the core of our grain detection module, demonstrated superior performance in terms of both detection accuracy (mAP50 = 99.10%) and inference speed (154 FPS) compared to other standard models. This highlights its potential for real-time or near-real-time applications. However, this enhanced performance comes with relatively high computational resource requirements, which presents a challenge for deploying the model on low-power, embedded agricultural devices at the edge. Future work could explore model compression techniques, such as pruning and quantization (Deng et al., 2020), to create a lightweight version of OPG-YOLOv8 without a significant loss of accuracy, thereby broadening its applicability.

### 4.3 Toward integrated and field-ready phenotyping

The current research successfully demonstrates the integration of multi-trait extraction within a single platform. However, the analysis of each trait is still largely independent. A promising future direction is to explore multi-task learning frameworks (Ruder, 2017), where a single model is trained to predict all traits simultaneously. Such an approach could leverage the inherent correlations between traits (e.g., panicle length and grain number) to improve the overall accuracy and efficiency of the system.

Ultimately, the goal is to move from controlled, laboratory environments to real-world field conditions (Tsaftaris and Scharr, 2019). This will require significant efforts in collecting diverse, field-based datasets and developing algorithms robust to challenges like variable illumination, complex backgrounds, and occlusion from leaves. Integrating multi-modal data, such as depth information from 3D sensors, could also be a key step in overcoming these hurdles. By addressing these challenges, this technology can be further enhanced as a powerful decision-support tool, helping to accelerate the development of improved rice varieties and contribute to global food security.

## 5 Conclusion

In this study, a precise and automated model for rice panicle trait extraction based on deep learning was successfully constructed, and a corresponding web-based system was developed. This work effectively addresses the critical need for efficient and high-throughput phenotyping in rice breeding and production. The primary innovations and conclusions of this research are summarized as follows:

1. A high-accuracy, multi-trait extraction pipeline was established. The proposed deep learning pipeline demonstrated strong performance in quantifying key agronomic traits. Notably, the panicle length extraction method achieved a high R² of 0.9583. For grain counting, a novel two-stage method combining density classification and regression modeling effectively corrected for occlusion errors, achieving R² values up to 0.9799 and significantly improving accuracy over direct detection.
2. An optimized object detection model (OPG-YOLOv8) was developed for superior performance. By integrating an attention mechanism (CBAM) and multi-scale fusion, the OPG-YOLOv8 model achieved an excellent balance of accuracy (mAP50 of 99.10%), computational efficiency (113.2G FLOPs), and speed (154 FPS), outperforming other comparative models. This provides a robust and efficient core engine for grain-level analysis.
3. An integrated and practical phenotyping system was implemented. The algorithms were encapsulated in a user-friendly, web-based “Rice Panicle Trait Extraction System.” This system translates complex computer vision models into a practical tool for breeders, bridging the gap between advanced research and real-world application by providing an end-to-end workflow from image upload to data export.

While this study has achieved its primary objectives, future work should focus on enhancing the model’s adaptability to complex field environments and further exploring the relationships among traits to develop more comprehensive and universally applicable phenotyping models. In summary, this research provides a powerful and efficient tool that can significantly promote the intelligent and digital breeding process of rice.

## Data Availability

The original contributions presented in the study are included in the article/supplementary material. Further inquiries can be directed to the corresponding authors.
